# Supplementary material for: Ornithine Aspartate and Vitamin-E Combination Has Beneficial Effects on Cardiovascular Risk Factors in an Animal Model of Nonalcoholic Fatty Liver Disease in Rats
Source: Biomolecules. 2022 Nov 28;12(12):1773. doi: 10.3390/biom12121773 (PMC9775092; doi:10.3390/biom12121773)
Supplement: Supplementary file 1 [file biomolecules-12-01773-s001.zip › biomolecules-1923750-supplementary.pdf]

Supplementary Data

Materials and Methods

Probes Identification for Gene Expression

Supplementary Table-S1 describes the identification of TaqMan probes that were used to evaluate the gene expression of circulating microRNAs.

Supplementary Table-S1: Probes identification

**Assay Name**	**Assay ID**
microRNAs	
rno-miR-122-5p	002245
hsa-miR-146a-5p	000468
hsa-miR-33a-5p	002135
hsa-miR-126-3p	002228
mmu-miR-499-5p	001352
hsa-miR-186-5p	002285
cel-miR-39-3p	000200

Results

General Characteristics

The general characteristics of the experimental model developed are shown in the Supplementary Table S2.

Supplementary Table-S2: General characteristics of the nutritional model of nonalcoholic fatty liver disease

Variables[#]	Control	NAFLD	NAFLD+LOLA	NAFLD+VitE	NAFLD+LOLA + VitE	p^*
Body weight (g)	180.9 (± 37.4) [a]	325.1 (± 92.2) [b]	310.0 (± 56.1) [b]	295.7 (± 90.5) [b]	332.7 (± 32.9) [b]	<0.001
Fresh liver weight (g)	17.2 (± 2.4) [a]	30.8 (± 4.3) [b]	27.3 (± 2.6) [b]	28.01 (± 4.9) [b]	30.3 (± 2.7) [b]	<0.001
Heart weight (g)	1.6 (± 0.04)	1.7 (± 0.07)	1.8 (± 0.05)	1.7 (± 0.05)	1.8 (± 0.04)	0.130
AST (U/L)	39.4 (± 7.5) [a]	80.0 (± 16.0) [b]	50.9 (± 18.2) [a]	54.0 (± 12.3) [a]	53.4 (± 19.8) [a]	<0.001
ALT (U/L)	81.0 (72.0-101.0) [a]	95.6 (70.8-177.5) [b]	97.0 (87-120.8) [a]	109.5 (79.5-129.8) [a]	112.0 (49.5-85.5) [a]	<0.001

[#] Variables expressed by mean (± standard deviation), or median (25th–75th percentiles). * $p < 0.05$ is considered significant, different letters indicate a significant difference between groups. Abbreviations: ALT: alanine aminotransferase; AST: aspartate; LOLA: ornithine aspartate; NAFLD: nonalcoholic fatty liver disease; and VitE: vitamin E.

Cardiomyocytes Histological Evaluation

The characterization of cardiomyocytes' morphometry (i.e., size and shape) of the experimental groups under study.

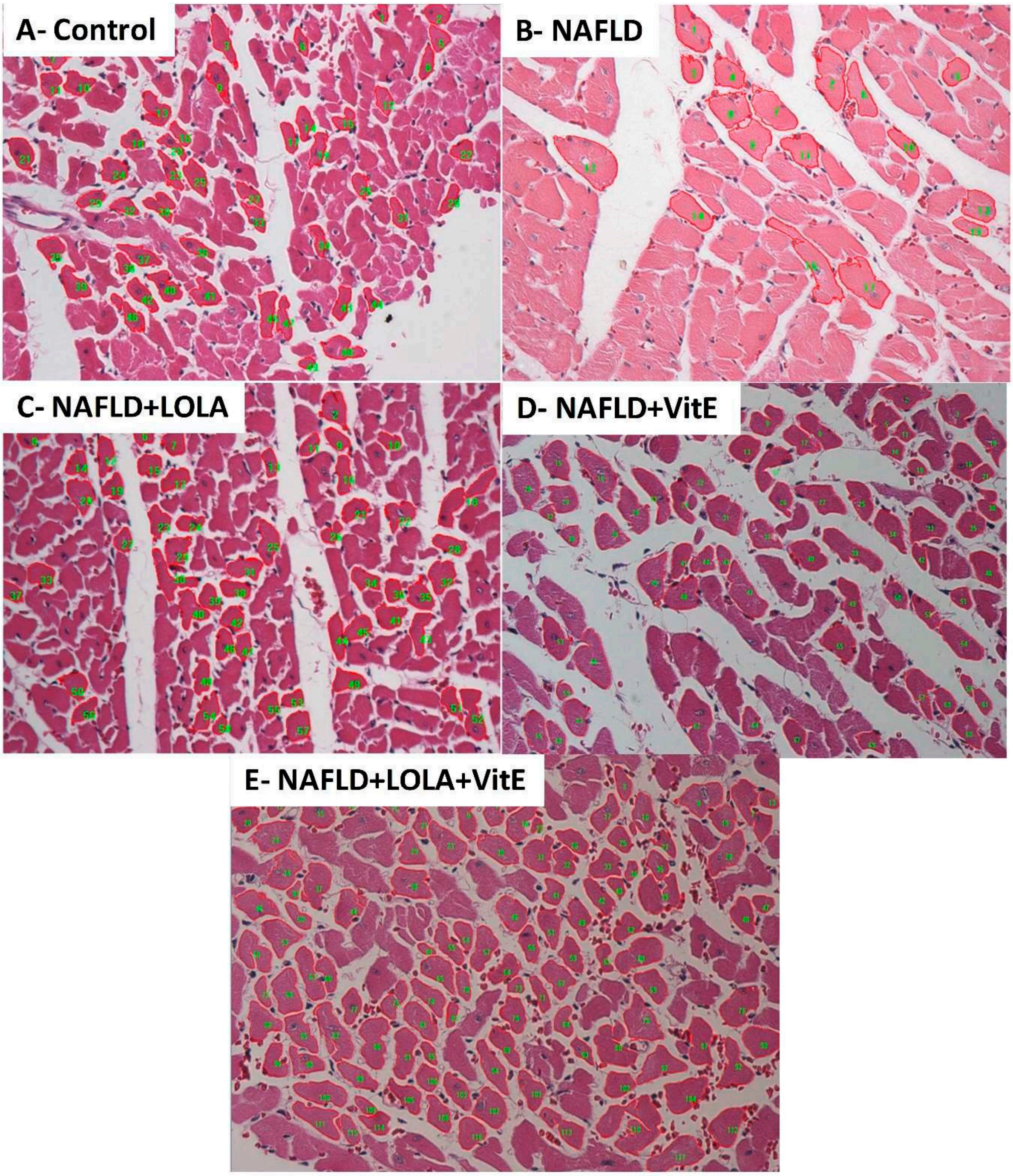

Figure S1: Cardiomyocytes histological evaluation. Images referring to the control (A), NAFLD (B), NAFLD + LOLA (C), NAFLD + VitE (D) and NAFLD + LOLA + VitE (E) groups. All images are stained with H&E. Panels with 40X magnification. LOLA: ornithine aspartate; NAFLD: nonalcoholic fatty liver disease and VitE: vitamin E.

Liver Histopathological Analysis

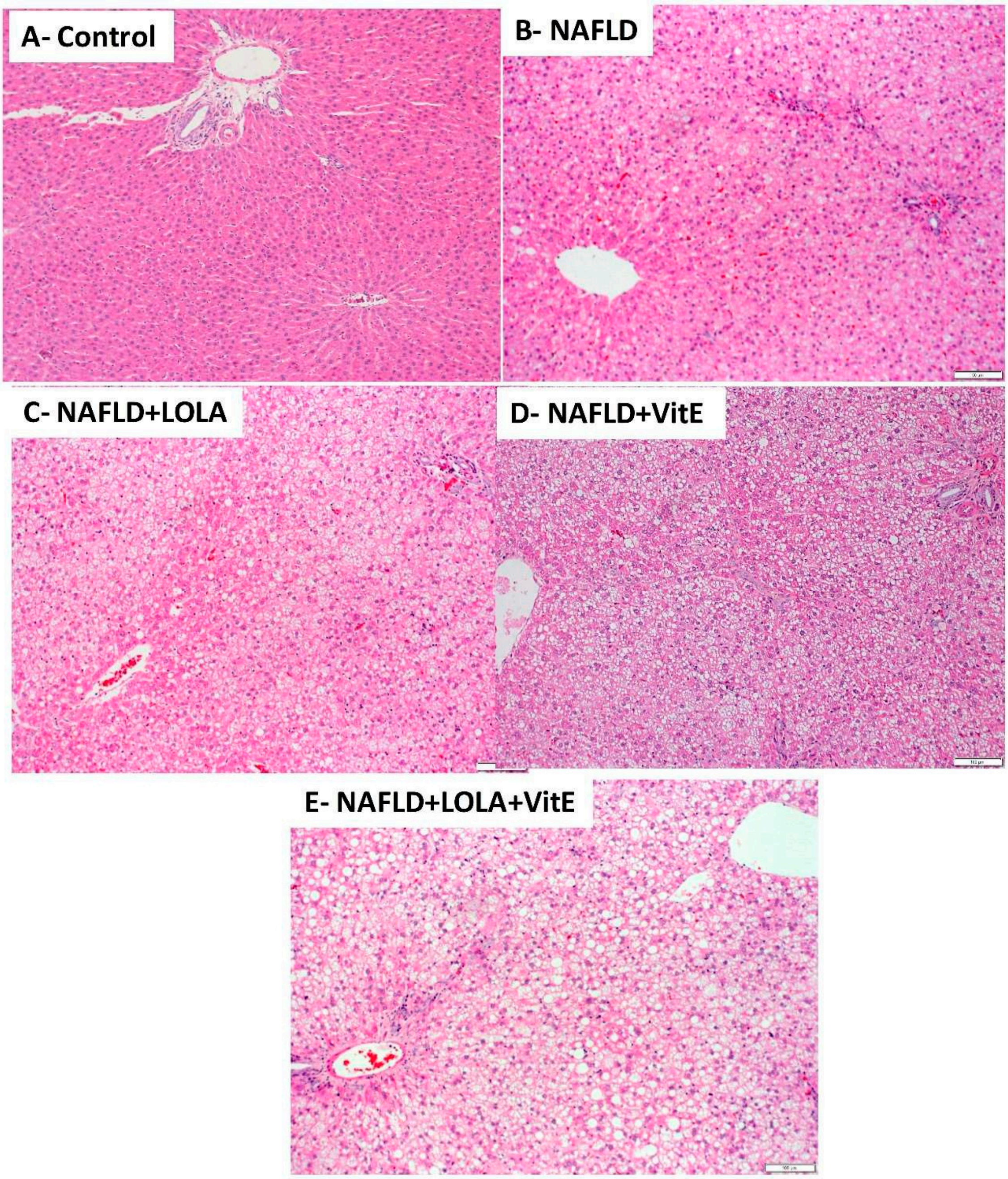

No abnormalities were observed in the liver tissue of the control group animals (Supplementary Figure S2A and Supplementary Figure S3A), whereas the animals in the NAFLD, LOLA, VitE, and LOLA+VitE groups (Supplementary Figure S2B–E and Supplementary Figure S3B–E) had predominant microvesicular steatosis along with moderate macrovesicular steatosis, inflammatory activity, mild hypertrophy and collagen fiber deposition.

Figure S2: Liver histological evaluation. Images referring to the control (A), NAFLD (B), NAFLD + LOLA (C), NAFLD + VitE (D) and NAFLD + LOLA + VitE (E) groups. All images are stained with H&E and the panels with 20X magnification. LOLA: ornithine aspartate; NAFLD: nonalcoholic fatty liver disease and VitE: vitamin E.

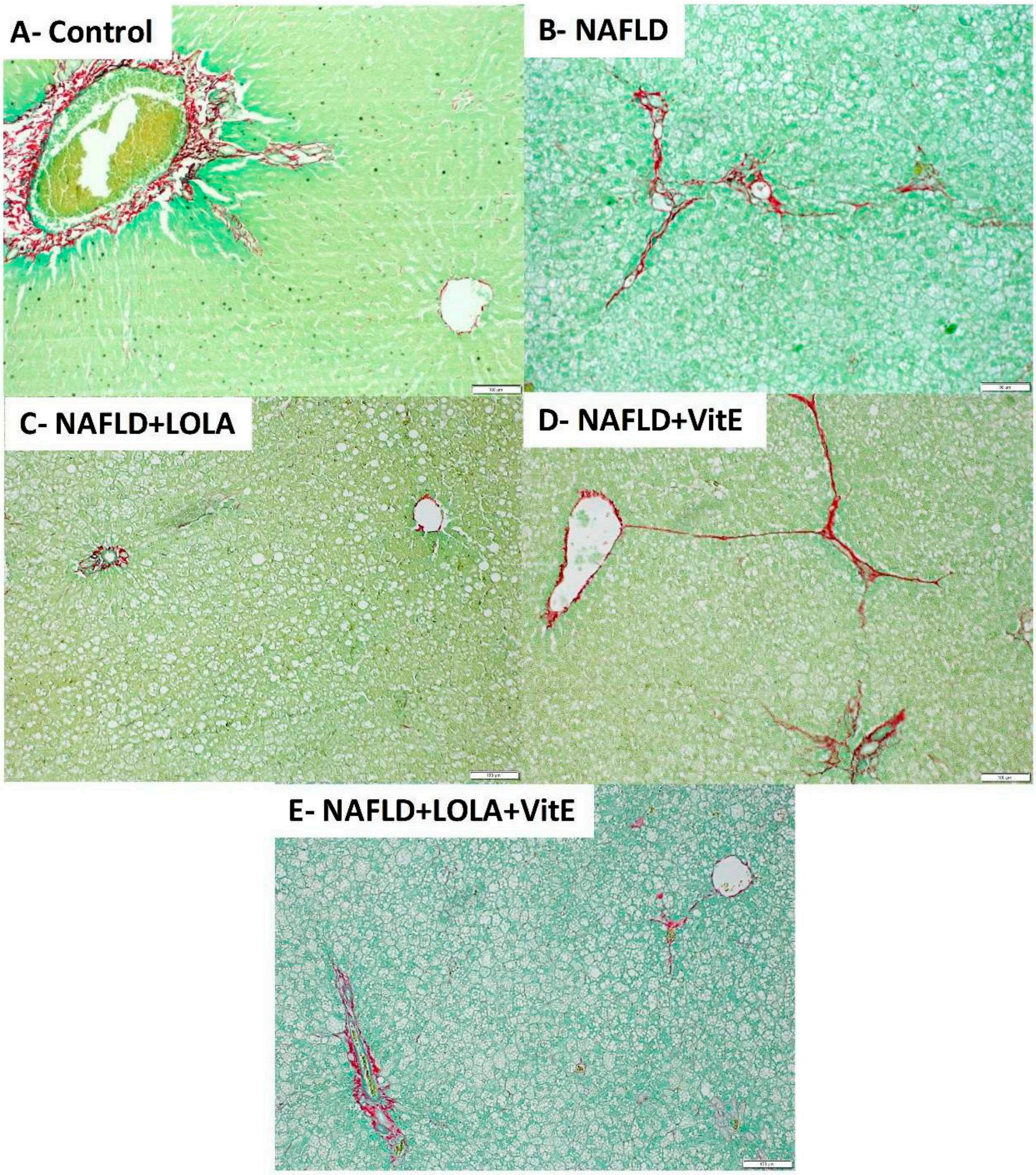

Figure S3: Liver histological evaluation. Images referring to the control (A), NAFLD (B), NAFLD + LOLA (C), NAFLD + VitE (D) and NAFLD + LOLA + VitE (E) groups, picrosirius staining and all panels with 20X magnification. LOLA: ornithine aspartate; NAFLD: nonalcoholic fatty liver disease and VitE: vitamin E.
